# Draft Genome Sequence of a *Neisseria meningitidis* Serogroup C Isolate of Sequence Type 11 Linked to an Outbreak among Men Who Have Sex with Men

**DOI:** 10.1128/genomeA.00795-13

**Published:** 2013-10-03

**Authors:** Frédéric J. Veyrier, Eva Hong, Ala-Eddine Deghmane, Muhamed-Kheir Taha

**Affiliations:** Institut Pasteur, Invasive Bacterial Infections Unit and National Reference Centre for Meningococci, Paris, France

## Abstract

Meningococcal disease occurs as sporadic cases in developed countries, with the occasional emergence of new clones of *Neisseria meningitidis*. Here, we report the genome sequence of *N. meningitidis* strain LNP27256, an isolate of sequence type 11 linked to a recent outbreak among men who have sex with men in Europe.

## GENOME ANNOUNCEMENT

*Neisseria meningitidis*, a Gram-negative human-specific bacterium, is the agent of severe and life-threatening invasive infections (mainly meningitis and/or septicemia). The incidence of meningococcal disease is region specific (1). However, meningococcal disease is still a major problem in developed countries due to frequent outbreaks that require prompt management, including public health measures (vaccination and chemoprophylaxis). Moreover, major epidemics occur in developing countries, such as those in sub-Saharan Africa. *N. meningitidis* is highly variable due to frequent genetic variations that occur through horizontal DNA exchanges (2). The reports on the increase of meningococcal disease incidence among men who have sex with men (MSM) have raised the question of whether a new clone is emerging in this community (3–5). However, the cases were due to isolates of serogroup C belonging to the clonal complex sequence type 11 (ST11), a genotype that is frequent among cases in the general population. Draft sequencing of isolates from MSM is therefore essential to compare the meningococcal isolates from that population to other isolates from the general population and should prompt a pathophysiological analysis of these isolates.

We announce the draft genome sequence of *N. meningitidis* strain LNP27256, isolated from a blood specimen at the Centre National Recherche Medicale (CNRM) at the Pasteur Institute, Paris, France, from a patient who died of meningitis. Initially, identification of the bacterium to the strain level and confirmation of the identification were carried out by conventional biochemical identification methods, as well as by multilocus sequence typing (MLST). Genomic DNA was extracted using the Genomic-tip 20/G kit (Qiagen) from an overnight culture grown on G2 plates. Whole-genome sequencing was performed using the Illumina HiSeq 2000 sequencer, which generated 100-bp paired reads. The sequencing was done by GATC Biotech using standard protocols as per the manufacturer’s instructions. The sequences (8 million reads) were *de novo* assembled using SeqMan NGen (DNAStar, Inc.) using default settings, wherein 223 contigs with 315× average genome coverage were obtained. The contig N_50_ was 20 kb, with an average length of 9,775. The contigs were submitted to the NCBI whole-genome shotgun (WGS) submission portal, and the sequences were annotated using the NCBI Prokaryotic Genome Automatic Annotation Pipeline (PGAAP) (http://www.ncbi.nlm.nih.gov/genomes/static/Pipeline.html).

A more-detailed comparative analysis of this genome with those of other *N. meningitidis* strains will provide further insight into the specific properties related to this strain that are linked to the recent outbreak among men who have sex with men in Europe.

### Nucleotide sequence accession numbers.

This whole-genome shotgun project has been deposited at DDBJ/EMBL/GenBank under the accession no. AVOU00000000. The version described in this paper is version AVOU01000000.
